# High MCM3 expression is an independent biomarker of poor prognosis and correlates with reduced RBM3 expression in a prospective cohort of malignant melanoma

**DOI:** 10.1186/1746-1596-7-82

**Published:** 2012-07-17

**Authors:** Björn Nodin, Marie Fridberg, Liv Jonsson, Julia Bergman, Mathias Uhlén, Karin Jirström

**Affiliations:** 1 Department of Clinical Sciences, Division of Pathology, Lund University, Skåne University Hospital, 221 85, Lund, Sweden; 2 Department of Immunology, Genetics and Pathology, Uppsala University, 751 85, Uppsala, Sweden; 3 Science for Life Laboratory, AlbaNova University Center, Royal Institute of Technology, 106 91, Stockholm, Sweden; 4 School of Biotechnology, AlbaNova University Center, Royal Institute of Technology, 106 91, Stockholm, Sweden

## Abstract

**Background:**

Malignant melanoma is the most lethal form of skin cancer with a variable clinical course even in patients with thin melanomas and localized disease. Despite increasing insights into melanoma biology, no prognostic biomarkers have yet been incorporated into clinical protocols. Reduced expression of the RNA binding motif protein 3 (RBM3) has been shown to correlate with tumour progression and poor prognosis in melanoma and several other cancer forms. In ovarian cancer, an inverse association was found between expression of RBM3 and the minichromosome maintenance 3 (MCM3) gene and protein. In melanoma, gene expression analysis and immunohistochemical validation has uncovered MCM3 as a putative prognostic biomarker. The aim of the present study was to examine the associations of MCM3 expression with clinical outcome and RBM3 expression in a prospective, population-based cohort of melanoma.

**Methods:**

Immunohistochemical MCM3 expression was examined in 224 incident cases of primary melanoma from the Malmö Diet and Cancer Study, previously analysed for RBM3 expression. Spearman´s Rho and Chi-Square tests were used to explore correlations between MCM3 expression, clinicopathological factors, and expression of RBM3 and Ki67. Kaplan Meier analysis, the log rank test, and univariable and multivariable Cox proportional hazards modelling were used to assess the impact of MCM3 expression on disease-free survival (DFS) and melanoma-specific survival (MSS).

**Results:**

High MCM3 expression was significantly associated with unfavourable clinicopathological features and high Ki67 expression. A significant inverse correlation was seen between expression of MCM3 and RBM3 (p = 0.025). High MCM3 expression was associated with a reduced DFS (HR = 5.62) and MSS (HR = 6.03), and these associations remained significant in multivariable analysis, adjusted for all other factors (HR = 5.01 for DFS and HR = 4.96 for MSS). RBM3 expression remained an independent prognostic factor for MSS but not DFS in the multivariable model.

**Conclusions:**

These findings provide validation of the utility of MCM3 expression as an independent biomarker for prognostication of patients with primary melanoma. Moreover, the inverse association and prognostic impact of MCM3 and RBM3 expression indicate a possible interaction of these proteins in melanoma progression, the functional basis for which merits further study.

**Virtual Slides:**

The virtual slide(s) for this article can be found here: http://www.diagnosticpathology.diagnomx.eu/vs/1814908129755401

## Introduction

Malignant melanoma is an aggressive form of cancer with an increasing incidence and mortality worldwide [1]. Once a patient has moved into the stage of generalized disease, survival is very poor [2,3], but the clinical course of melanoma is highly variable even in patients with thin melanomas and localized disease [4-6]. Despite increasing insights into melanoma biology and advances in various “omics” technologies [7-9], no prognostic biomarkers have yet been incorporated into clinical protocols. We have previously demonstrated that high nuclear expression of the RNA binding motif protein 3 (RBM3) is associated with an improved outcome in several major cancer forms, i.e. breast, ovarian, colorectal and prostate cancer and malignant melanoma [10-14]. In malignant melanoma, a significantly downregulated expression of RBM3 was observed in metastases compared to primary melanoma [12], which is in line with previous *in vitro* data demonstrating a significant downregulation of RBM3 expression in metastatic compared to primary melanoma cells [15]. The functional basis for the observed associations of loss of RBM3 expression with tumour progression and poor prognosis in human cancer remains largely unclear, but in epithelial ovarian cancer (EOC), the association of RBM3 expression and improved outcome has to some extent been corroborated by *in vitro* data demonstrating an association between RBM3 expression and improved response to cisplatin treatment [11]. Moreover, gene set enrichment analysis (GSEA) in human EOC has revealed an association between RBM3 expression and several processes involved in maintenance of DNA integrity and repair[16]. Specifically, an inverse association was observed between expression of RBM3 and the minichromosome maintenance 3 (MCM3) gene and protein in human EOC samples and in ovarian cancer cells, and high expression of MCM3 was also demonstrated to be associated with poor prognosis in EOC, both at the mRNA and protein levels [16]. MCM proteins (MCM2-7) constitute a family of highly conserved DNA-binding proteins with essential functions in the initiation and regulation of DNA replication [17,18]. In malignant melanoma, gene expression analysis and independent immunohistochemical validation have uncovered several MCM family members, i.e. MCM3, MCM4 and MCM6 as biomarkers of poor prognosis [8]. MCM3 expression has also been asssociated with poor prognosis in malignant glioma [19] and medulloblastoma [20].

In light of the findings of an inverse association between expression of MCM3 and RBM3 in EOC, and their prognostic implications in melanoma, the aim of the present study was to examine the associations of immunohistochemical MCM3 expression with expression of RBM3, clinicopathological factors and survival in a prospective, population-based cohort of malignant melanoma (n = 224), previously analysed for RBM3 expression [12].

## Methods

### Patients

Until end of follow-up 31 December 2008, 264 incident cases of malignant melanoma had been registered in the prospective, population-based cohort study Malmö Diet and Cancer Study (MDCS) [21]. Cases were identified from the Swedish Cancer Registry up until 31 Dec 2007, and from The Southern Swedish Regional Tumour Registry for the period of 1 Jan-31 Dec 2008. Nine (3.4 %) cases for whom clinical and pathology records were missing were excluded from the study, leaving 255 cases available for analysis. All tumours with available slides and/or paraffin blocks were histopathologically re-evaluated on haematoxylin and eosin stained slides whereby information on lymphocytic infiltration (none, mild, moderate or brisk), ulceration (absent or present), mitotic count and vascular invasion was obtained. Data on location, Clark level and Breslow depth of invasion was obtained from the clinical- and/or pathology records. Information on recurrence (local, regional or distant) was obtained in 2010 from patient records and pathology reports. Information on vital status and cause of death was obtained from the Swedish Cause of Death Registry up until 31 Dec 2009. Follow-up started at date of diagnosis and ended at death, emigration or 31 Dec 2009, whichever came first. Median follow-up time was 6.84 years (range 0.64-17.05) for the full cohort (n = 255) and 7.29 years (range 1.10-17.05) for patients alive (n = 202). Patient and tumour characteristics of the cohort have been described in detail previously [12,22]. Ethical permission was obtained from the Ethics Committee at Lund University for the MDCS (Ref. 51/90), and the present study (Ref. 530/2008).

### Tissue microarray construction, immunohistochemistry and evaluation of MCM3 staining

Paraffin-embedded tumour specimens were collected from the archives of the pathology departments in the region of Skåne in southern Sweden. Tumours with an insufficient amount of material were excluded whereby 226/255 (88.6 %) cases were suitable for TMA construction. Areas representative of cancer were then marked on haematoxylin & eosin stained slides and TMAs constructed as previously described [12]. In brief, three 0,6 mm cores were taken from each tumour and mounted in a new recipient block using semi-automated arraying device (TMArrayer, Pathology Devices, Westminster, MD, USA). In addition, metastases (representing both regional and distant metastases in various organs) were sampled from 31 cases, for some of which 1.00 mm cores were used. Presence of melanoma in the TMA cores was ascertained with immunohistcohemical staining for Melan-A. For immunohistochemical analysis of MCM3 expression, 4 um TMA-sections were automatically pre-treated using the PT-link system (DAKO, Glostrup, Denmark) and then stained in an Autostainer Plus (DAKO) using a polyclonal MCM3-antibody (HPA004789, diluted 1:700). MCM3 is expressed in the nucleus and both the fraction of positive cells and staining intensity were taken into account. Nuclear fraction was categorized into four groups, namely 0 (0-1 %), 1 (2-25 %), 2 (26–75) and 3 (> 75 %) and nuclear staining intensity denoted as 0–3, whereby 0 = negative, 1 = intermediate, 2 = moderate and 3 = strong intensity. A combined nuclear score (NS) was then constructed as a multiplier of MCM3 nuclear fraction and intensity, thus ranging from 0 to 9. MCM3 expression was also evaluated on a subset of full-face sections (n = 20). Ovarian cancer samples known to have high and negative MCM3 expression[16] were included as controls, as well as normal liver (negative control) and human tonsil (positive control) (http://www.proteinatlas.org). The immunohistochemical staining was evaluated by three independent observers (BN, MF and KJ), including one board certified pathologist (KJ), who were blinded to clinical and outcome data. Scoring differences were discussed in order to reach consensus.

Immunohistochemical staining and analysis of RBM3 and Ki67 expression was performed as previously described [12,22].

### Statistical analysis

Chi-square, Spearman´s Rho and Mann Whitney U tests were used for correlation analyses of MCM3 expression with clinicopathological characteristics and expression of RBM3 and Ki67. Disease-free survival (DFS) time was determined from the date of diagnosis of the primary melanoma to the date of diagnosis of the first local, regional or distant recurrence or death from malignant melanoma. Follow-up started at date of diagnosis and ended at recurrent disease, death, lost to follow-up (emigration) or last date of follow-up with regard to recurrent disease. No recurrences were recorded following the last date of follow-up regarding death, i.e. 31 Dec 2009. The kappa-test was used to compare the reliability of TMA-based vs full-face section based scoring of MCM3. Kaplan-Meier analysis and log rank test were used to illustrate differences in DFS and melanoma-specific survival (MSS). Cox regression proportional hazards models were used to estimate the impact of the investigated parameters on DFS and MSS in both uni- and multivariable analysis. Some subjects had no information on one or several markers and missing values were coded as a separate category for categorical variables and as the mean of all observations for continuous variables. Missing values for categorical variables co-varied and the multivariable model did not converge due to many constant values. In order to avoid this, the multivariable analysis only included patients with information on MCM3 expression. Co-variates were entered into the multivariable analysis using backward selection where a p-value of 0.05 decided entry and a p-value of 0.10 was used for removal. All calculations were performed using IBM SPSS Statistics Version 20 (SPSS Inc, Chicago, IL). All statistical tests were two-sided and a p value < 0.05 was considered statistically significant.

## Results

### Distribution of MCM3 expression in primary and metastatic melanoma

**Figure 1:**
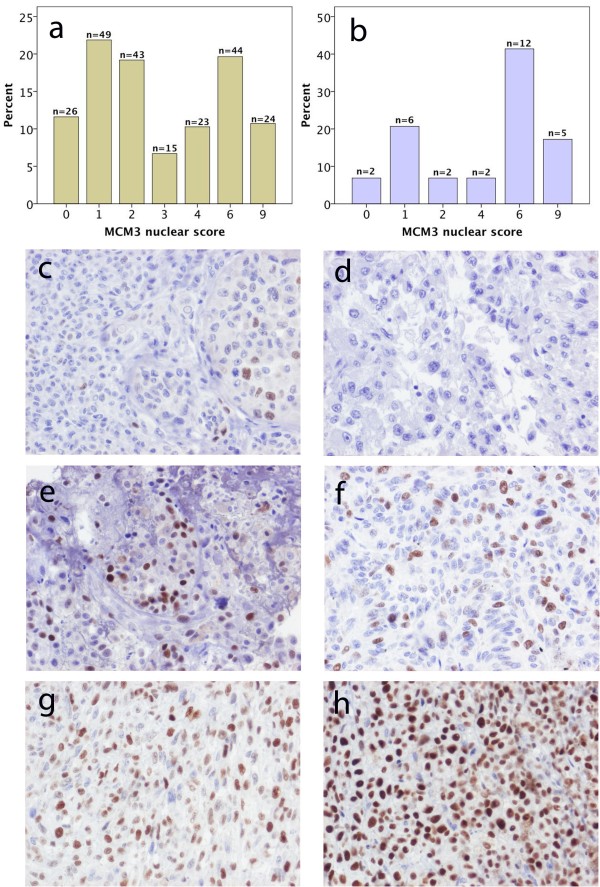
**Distribution and sample immunohistochemical images of MCM3 expression in primary and metastatic melanoma.** Distribution of immunohistochemical MCM3 expression denoted as the multiplier of fraction and intensity of staining (nuclear score = NS) in **a**) primary melanoma and **b**) metastases. Sample images of MCM3 expression in primary tumour and paired metachronous metastasis: **c**) nodular melanoma on left ear (NS = 2) and **d**) lymph node metastasis in left groin (NS = 0); **e**) superficial spreading melanoma on lower left leg (NS = 6) and **g**) cutaneous metastasis on back of neck (NS = 4); nodular melanoma on lower right leg (NS = 6) and cutaneous metastasis on lower right leg (NS = 9).

MCM3 expression could be evaluated in 224/226 (99.1 %) primary tumours and 29/31 (93.5 %) metastases. The non-evaluated cases did not contain sufficient amount of tumour. Twentysix (11.6 %) primary tumours and 2 (6.9 %) metastases lacked RBM3 expression and in the remaining cases, MCM3 was expressed in various fractions and intensities as demonstrated by the distribution of the nuclear score in Figure 1a and b. In contrast to RBM3 [12], there was no significant difference in MCM3 expression (nuclear score) in paired primary melanoma and metastatic lesions (Spearman´s Rho = 0.24; p = 0.205). There was an excellent correlation betweeen estimation of the nuclear score in TMA-cores and full-face sections (kappa-value: 0.89). Sample immunohistochemical images of primary tumours and paired metachronous metastases are shown in Figure 1c-h.

### Prognostic value of MCM3 expression and associaton with clinicopathological factors

**Figure 2:**
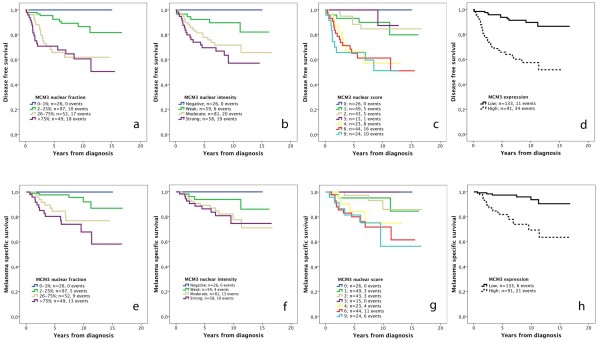
**Kaplan-Meier estimates of the impact of MCM3 expression on disease free and melanoma-specific survival.** Disease survival in strata according to (**a**) the fraction of staining, logrank p <0.001, (**b**) intensity of staining, logrank p =0.001, (**c**) nuclear score, logrank p < 0.001, and (**d**) low vs high staining, logrank p < 0.001. Melanoma-specific survival in strata according to (**e**) the fraction of staining, logrank p <0.001, (**f**) intensity of staining, logrank p = 0.053, (**g**) nuclear score, logrank p < 0.003, and (**h**) high vs low staining, logrank p < 0.001. The nuclear score refers to a multiplier of fraction and intensity. Low MCM3 expression refers to tumours with a nuclear score ≤3 and high expression to tumours with a nuclear score >3.

Next, we examined the prognostic value of MCM3 expression in strata according to the nuclear fraction, nuclear intensity, nuclear score and a dichotomized variable of low (NS < =3) and high (NS > 3) MCM3 expression. As demonstrated in Figure 2, Kaplan-Meier analysis revealed a stepwise decreased DFS and MSS with increasing fractions and intensities of MCM3 expression (Figure 2a, b, e, f). Survival analysis in strata according to NS revealed a similar prognosis for patients with tumours having a NS < =3 and >3, respectively (Figure 2c, g), thus forming basis for selection of cutoff for a dichotomized variable of low (NS > =3) vs high (NS > 3) MCM3 expression (Figure 2d, h). Notably, none of the patients with melanomas lacking MCM3 expression had recurrent disease or died from melanoma.

Associations between low and high MCM3 expression, established prognostic factors and RBM3 expression are shown in Table 1. High MCM3 expression was significantly associated with unfavourable clinicopathological features, i.e. nodular subtype (p = 0.003), Breslow thickness (p < 0.001), Clark level (p < 0.001), clinical stage (p = 0.003), vascular invasion (p = 0.016), mitotic count (p < 0.001), and high Ki67 expression (p = 0.001). A significant inverse association was seen between expression of MCM3 and RBM3 (p = 0.025). In metastatic melanoma, no significant association was observed between expression of MCM3 and RBM3 (data not shown). There was no significant association between RBM3 and Ki67 expression in primary melanomas (data not shown).

**Table 1:** Associations between MCM3 expression and clinicopathological factors

	***MCM3 expression***		
***Factor***	***Low***	***High***	***P***
n(%)	133 (59.4)	91 (40.6)	
**Age (continuous)**			*0.858*
Mean	67.36	67.25	
Median	69.00	69.00	
Range	46-81	47-83	
**Location**			*0.893*
Dorsal trunk	33 (26.0)	23 (25.6)	
Frontal trunk	16 (12.6)	12 (13.3)	
Arms	22 (17.3)	18 (20.0)	
Legs	37 (29.1)	23 (25.6)	
Head and neck	19 (15.0)	14 (15.6)	
*Unknown*	*6*	*1*	
**Histological subtype**			*0.003*
SSM	89 (66.9)	53 (58.9)	
LMM	18 (13.5)	5 (5.6)	
NMM	23 (17.3)	30 (33.3)	
Other	3 (2.3)	2 (2.2)	
*Unknown*		*1*	
**Breslow thickness**			*<0.001*
Mean	1.48	1.94	
Median	0.55	1.05	
Range	0.08-40	0.15-14	
*Unknown*	*1*	*1*	
**Breslow categories**			*<0.001*
≤ 1 mm	93 (70.5)	44 (48.9)	
1.1-2.0 mm	19 (14.4)	15 (16.7)	
2.1-4.0 mm	14 (10.6)	23 (25.6)	
> 4.0 mm	6 (4.5)	8 (8.9)	
*Unknown*	*1*	*1*	
**Clark level**			*<0.001*
II	58 (43.9)	19 (21.3)	
III	58 (43.9)	39 (43.8)	
IV-V	16 (12.1)	31 (34.8)	
*Unknown*	*1*	*2*	
**Ulceration**			*<0.001*
No	122 (91.7)	67 (74.4)	
Yes	11 (8.3)	23 (25.6)	
*Unknown*		*1*	
**Clinical stage**			*0.003*
1	108 (91.5)	50 (75.8)	
2-4	10 (8.6)	16 (24.2)	
*Unknown*	*15*	*24*	
**Vascular invasion**			*0.016*
No	129 (97.0)	81 (89.0)	
Yes	4 (3.0)	10 (11.0)	
**Mitotic count**			*<0.001*
0/mm^2^	87 (65.4)	27 (29.7)	
> = 1/mm^2^	46 (34.6)	64 (70.3)	
**Lymphocytic infiltrate**			*0.463*
None-mild	40 (30.5)	23 (25.6)	
Moderate-brisk	93 (69.9)	67 (74.4)	
*Unknown*		*1*	
**Ki67 expression**			*0.001*
Low	100 (85.5)	58 (65.9)	
High	17 (14.5)	30 (34.1)	
*Unknown*	*16*	*3*	
**RBM3 expression**			*0.025*
Low	46 (37.4)	48 (52.7)	
High	77 (62.6)	43 (47.3)	
*Unknown*	10	0	

P-values refer to Mann Whitney U test for comparison of medians and Chi square test for X × 2 tables. The categories marked as unknown were not included in the analysis.

Low RBM3 expression corresponds to a nuclear staining intensity of negative to moderate and high expression corresponds to strong staining as previously described [12].

The prognostic value of MCM3 was confirmed in univariable Cox regression analysis (Table 2) and remained significant in a multivariable model, adjusted for clinicopathological factors, Ki67 and RBM3 expression (Table 2). Notably, while only MCM3 expression remained prognostic for DFS, both high MCM3 expression and low RBM3 expression remained independent factors of a shorter MSS. MCM3 expression was associated with a significantly reduced overall survival (OS) in univariable analysis, but not in multivariable analysis (data not shown), and in contrast to our previous study [12], the prognostic impact of RBM3 expression on OS was lost when MCM3 was included in the model (data not shown). The prognostic value of MCM3 did not differ according to gender (data not shown).

**Table 2:** Relative risks of recurrent disease and death from melanoma according to MCM3 expression

		**Risk of disease recurrence**			**Risk of death from melanoma**	
		**Univariable**	**Multivariable**		**Univariable**	**Multivariable**
	**n (events)**	**HR (95%CI)**	**HR (95%CI)**	**n (events)**	**HR (95%CI)**	**HR (95%CI)**
**Age**						
Continuous	255 (47)	1.01 (0.97-1.05)		255 (28)	1.05 (0.99-1.10)	1.08 (1.02-1.14)
**Gender**						
Female	132 (21)	1,00		132 (9)	1,00	
Male	123 (26)	1.51 (0.85-2.70)		123 (19)	2.77 (1.25-6.14)	
**Clark level**						
II	93 (5)	1,00		93 (2)	1,00	
III	103 (21)	4.39 (1.65-11.65)		103 (14)	7.16 (1.63-31.50)	
IV-V	51 (21)	9.99 (3.76-26.55)		51 (12)	12.91 (2.89-57.74)	
**Breslow**						
Continuous	248 (47)	1.07 (1.03-1.11)		248 (28)	1.08 (1.03-1.13)	
**Subtype**						
SSM, LMM, Other	195 (22)	1,00		195 (11)	1,00	1.00
Nodular	53 (24)	5.63 (3.15-10.08)		53 (16)	7.19 (3.32-15.57)	5.51 (2.39-12.71)
**Ulceration**						
No	214 (30)	1,00		214 (18)	1,00	
Yes	35 (16)	5.81 (3.13-10.80)		35 (9)	5.73 (2.59-12.97)	
**Lymphocytic**						
**infiltrate**						
0-1	72 (20)	1,00		72 (11)	1,00	
2-3	176 (26)	0.43 (0.24-0.78)		176 (16)	0.50 (0.23-1.08)	
**Clinical stage**						
I	179 (12)	1,00	1,00	179 (7)	1,00	
II-IV	28 (13)	15.02 (6.39-35.30)	12.03 (4.49-32.28)	28 (8)	14.09 (4.82-41.18)	
**Mitotic count**						
<1/mm^2^	131 (7)	1,00		131 (3)	1,00	
> = 1/mm^2^	122 (40)	7.96 (3.56-17.80)		122 (25)	11.25 (3.39-37.36)	
**Vascular invasion**						
No	232 (35)	1,00	1,00	232 (18)	1,00	1.00
Yes	14 (11)	9.25 (1.67-7.60)	3.56 (2.35-10.92)	14 (9)	11.29 (5.05-25.25)	8.26 (3.30-20.67)
**Ki67 expression**						
Low (<=25 %)	159 (28)	1,00		159 (16)	1,00	
High (>25 %)	47 (16)	2.46 (1.33-4.56)		47 (10)	2.64 (1.20-5.83)	
**RBM3 expression**						
Low	95 (24)	1.00		95 (17)	1.00	1.00
High	120 (20)	0.50 (0.27-0.91)		120(9)	0.29 (0.13-0.66)	0.30 (0.14-0.71)
**MCM3 expression**						
Low (N ≤ S3)	133 (11)	1.00	1.00	133 (6)	1.00	1.00
High (NS > 3)	91 (34)	5.62 (2.83-11.13)	5.01 (0.33-10.79)	91 (21)	6.03 (2.42-15.02)	4.96 (1.77-13.87)

Low RBM3 expression corresponds to a nuclear staining intensity of negative to moderate and high expression corresponds to strong staining as previously described [12]. The number of cases in the multivariable analysis is equal to the number of cases evaluated for MCM3 expression (n = 224).

**Figure 3:**
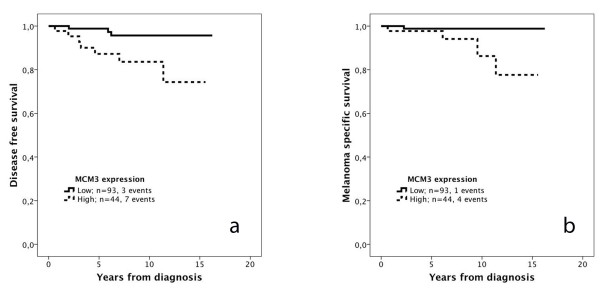
**Kaplan-Meier estimates of the impact of MCM3 expression on disease free and melanoma-specific survival in thin melanomas.** The impact of low and high MCM3 expression on (**a**) disease free survival, logrank p = 0.007, and (**b**) melanoma-specific survival, logrank p = 0.031, in thin melanoma (<= 1 mm).

The prognostic value of MCM3 expression in thin melanomas (≤ 1 mm; n = 137) was also examined. As visualized in Figure 3, Kaplan Meier analysis revealed a significant association between high MCM3 expression and a reduced DFS (Figure 3a) and MSS (Figure 3b). These associations were confirmed in univariable Cox regression analysis for both DFS (HR = 5.23, 95 % CI 1.35-19.23) and MSS (HR = 9.63, 95 % CI 1.07-86.44). RBM3 expression has previously been shown not to be prognostic in thin melanomas ≤ 1 mm [12]. Due to the small number of events, multivariable analysis could not be performed for MSS, but for DFS, only nodular subtype remained an independent prognostic factor (HR = 6.37; 95 % CI 1.19-34.03) and MCM3 expression did not remain significant (HR = 3.74;95 % CI 0.85-16.41).

### Survival according to categories of MCM3 and RBM3 expression

**Figure 4:**
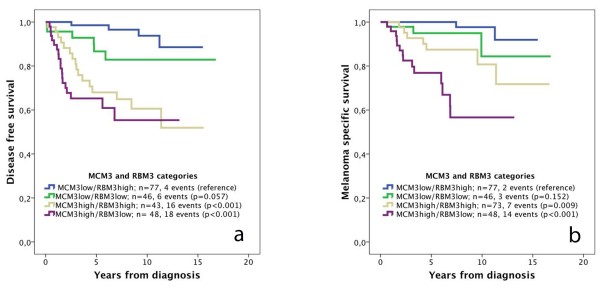
**Kaplan-Meier estimates of disease free and melanoma-specific survival in combined strata of high and low MCM3 and RBM3 expression.** The impact of combined categories low and high expression of MCM3 and RBM3 expression, respectively, on (**a**) disease free survival and (**b**) melanoma-specific survival.

In light of the observed inverse association between expression of MCM3 and RBM3 in primary melanoma, we also examined differences in survival according to combined categories of low and high MCM3 and RBM3 expression (Figure 4). Kaplan Meier analysis revealed that the combination of high MCM3/low RBM3 expression was associated with the poorest DFS (Figure 4a) and MSS (Figure 4b) and the combination of high MCM3/low RBM3 expression with the best survival, with somewhat differing outcome for patients with tumours expressing either low or high levels of both markers, depending on the survival endpoint.

## Discussion

In this study, we have demonstrated that high immunohistochemical expression of MCM3 is an independent predictor of an increased risk of recurrence and death from melanoma in a large, prospective, population-based cohort study. These findings are in line with previous results from gene expression analysis and immunohistochemical validation in retrospective melanoma cohorts, where MCM3, together with MCM4 and MCM6, was found to correlate with poor outcome, although only MCM4 and MCM6 remained independent predictors of survival in multivariable analysis [8]. While we are not aware of any other validatory studies on the prognostic value of MCM3 expression in melanoma, a recent study failed to confirm the prognostic value of MCM4 in a consecutive cohort of nodular melanoma (n = 220) [23]. Given the uniformity regarding histological subtype in that study, the results may however not be applicable to the general population. In our study, MCM3 expression was strongly associated with nearly all unfavourable clinicopathological characteristics and, yet, remained an independent predictor of a reduced DFS and MSS, adjusted for all other factors. As denoted and discussed previously, the proportion of thin melanomas in this cohort is higher than the expected [12], despite the higher age of participants in the MDCS (all > 40 years at study entry) and the fact that older melanoma patients more frequently present with advanced disease [24]. Nevertheless, as current clinical guidelines in Sweden recommend sentinel node biopsy only in melanomas > 1 mm, there is a great unmet need for identification of prognostic biomarkers in thin melanomas (≤ 1 mm) [25], not least since this category seems to make up for most of the increasing melanoma incidence [26]. Therefore, despite the possibility of a selection bias and the small number of events, the comparatively large proportion of thin melanomas in the MDCS may offer some advantage in biomarker studies. The study by Winnepenninckx et al. was limited to melanomas in the vertical growth phase and/or with a thickness of > 1 mm [8] and, notably, in our study, MCM3 expression was found to be prognostic also in the category of thin melanomas. Thus, the potential value of MCM3 expression as a prognostic biomarker in thin melanomas merits further investigation in larger patient cohorts.

A cautionary remark should also be made regarding methodological aspects on the use of the TMA technique for biomarker studies in malignant melanoma, mainly concerning the technical difficulty in sampling of small tumours. As previously pointed out, the distribution of clinicopathological characteristics was similar in tumours included in the TMA cohort (n = 226) and tumours not suitable for TMA construction (n = 29) in the full cohort, with exception for histological subtype, with no tumours being denoted as nodular in the category not suitable or available for TMA construction, but an equal distribution of the other subtypes [22]. Another limitation to the TMA technique is that it might not accurately reflect the expression of heterogenously expressed markers. Therefore, assessment of MCM3 expression was also performed in a subset of full-face sections, which resulted in a kappa-value corresponding to the best degree of concordance [27].

The here observed inverse association of MCM3 and RBM3, both regarding their tumour-specific expression and impact on survival, is in line with previous *in vivo* and *in vitro* observations in ovarian cancer [16]. Notably, MCM3 expression was an independent predictor of both DFS and MSS while RBM3 only remained an independent predictor of CSS and OS [12]. Longitudinal analysis did not reveal an altered expression of MCM3 in metastatic compared to primary melanoma, in contrast to RBM3, that was found to be downregulated in metastatic melanoma in two independent studies, including the present cohort [12,15]. Moreover, RBM3 expression was not found to be prognostic in thin melanomas, but an independent prognostic factor in melanomas thicker than 1 mm [12]. Expression of MCM3 but not RBM3 correlated significantly with Ki67 expression, which is in line with its role as a marker of proliferation [28,29]. In contrast to MCM3, however, Ki67 expression was not prognostic in the full cohort in our previous study, only in male melanoma [22]. Speculatively, these findings indicate that loss of RBM3 may be associated with a switch towards a more invasive and/or metastatic rather than proliferative phenotype, while up-regulated MCM3 expression may either be associated with increased proliferation or functions beyond DNA licensing, i.e. cell migration and invasion, as demonstrated in medulloblastoma cells [20].

In a translational context, while MCM3 expression appears to be a stronger prognostic biomarker than RBM3, not least in thin melanomas, the inverse correlation between tumour-specific expression of MCM3 and RBM3, which is in line with previous observations in ovarian cancer [16], may give some directions towards further functional studies of the role and interaction of these proteins in melanoma progression and metastasis. While both proteins have been demonstrated to be up-regulated in several premalignant conditions and cancer forms compared to their corresponding normal tissues [13,17,28-31], it is becoming increasingly evident that their oncogenic activities in human tumours influence clinical outcome in opposite ways. One explanation for this may be that elevated expression of RBM3 attenuates DNA damage response, in which MCM proteins play an important role [17,32], thus preventing the selection of clones with an increased capability for invasion and metastatic spread [33,34]. Along this line, it would also be of interest to study the expression of MCM3 and RBM3 in benign naevi, dysplastic naevi and melanoma in situ in order to assess their potential role as markers of genetic abnormalities and high-risk lesions, for which e.g. FISH testing has been identified as a valuable diagnostic tool [35]. Ladstein et al. compared MCM4 expression in benign naevi and melanoma and found significantly higher MCM4 positivity in melanoma compared to benign naevi [23].

## Conclusions

The findings from this prospective cohort study provide an independent validation of MCM3 expression as a biomarker of poor prognosis in malignant melanoma, also in the category of thin melanomas, which may be of particular clinical relevance. Moreover, we have demonstrated an inverse association between tumour-specific expression of MCM3 and the RBM3 protein, loss of which has previously been found to correlate with tumour progression and poor prognosis in melanoma. The mechanistic basis for these observations should be addressed in future studies.

## Abbreviations

MCM3, Minichromosome maintenance protein 3; RBM3, RNA-binding motif protein 3; DFS, Disease free survival; MSS, Melanoma-specific survival; MDCS, Malmö Diet and Cancer Study.

## Competing interests

The authors declare that no competing interests exist.

## Authors contributions

BN carried out and evaluated the immunohistochemical stainings, performed the statistical analyses and drafted the manuscript. MF evaluated the immunohistochemical stainings and helped draft the manuscript. LJ and LB collected clinical data. MU contributed to the conception and design of the study. KJ conceived of the study, carried out the histopathological re-evaluation, evaluated the immunohistochemistry, and helped draft the manuscript. All authors read and approved the final manuscript.
